# Depressive symptoms and associated factors in medical interns at a tertiary hospital

**DOI:** 10.4102/sajpsychiatry.v25i0.1322

**Published:** 2019-07-04

**Authors:** Kaveshin Naidu, John R. Torline, Michelle Henry, Helena B. Thornton

**Affiliations:** 1 Department of Psychiatry and Mental Health, Faculty of Health Sciences, University of Cape Town, Cape Town, South Africa; 2 Specialist Psychiatrist, Cape Town, South Africa; 3 Centre for Higher Education Development, University of Cape Town, Cape Town, South Africa

**Keywords:** Depressive symptoms, Burnout, Medical interns, Beck Depression Inventory 2, South Africa

## Abstract

**Background:**

It is known that medical doctors suffer from increased rates of depression with medical interns being most at risk. Despite this, little is known about the prevalence of depression in interns in South Africa.

**Objectives:**

This study aimed to assess the prevalence of depressive symptoms in interns employed at Groote Schuur Hospital, a tertiary hospital in the Western Cape.

**Method:**

The study was a cross-sectional study. All 91 interns were invited to participate in the study and consenting interns were required to complete a demographic and related questionnaire and the Beck Depression Inventory 2 (BDI-2).

**Results:**

Fifty-four (59.3%) of all invited interns participated in the study. Twenty-two interns (40.7%) reported a BDI-2 score of 14 or greater, indicating at least mild self-reported symptoms of depression. Features associated with a BDI-2 score of 14 or greater, included female gender, a previous diagnosis of depression, seeing a psychotherapist and previously being on antidepressant medication during internship. Other features also significantly associated with higher BDI-2 scores included suicidal ideation, thoughts of emigration, wanting to leave medicine and using substances to cope. The most significant associated feature of high BDI-2 scores was a subjective feeling of being ‘burnt out’.

**Conclusion:**

Interns had a higher prevalence of depressive symptoms when compared to the general population. The feeling of being ‘burnt out’ was the most significant factor associated with the severity of depressive symptoms. It is imperative that the mental health of both medical students and newly qualified doctors be prioritised, supported and monitored.

## Introduction

It is established that doctors suffer increased rates of depression and increasingly, it is being shown that medical interns (hereafter referenced as interns) are at even higher risk.^1,2,3,4,5,6,7,8,9,10,11,12,13^ In South Africa, the lifetime prevalence of depression in the general population is 9.7% with little known about the prevalence of depression in South African medical doctors including interns.^14^

In a 2015 meta-analysis published by Mata et al., the pooled prevalence of depressive symptoms in medical doctors was 28.8% with 20.9% – 43.2% of interns screening positive for depression.^2^ Most studies included in this meta-analysis were conducted in North America. One study that was conducted in Kenya showed that at least 62.0% of interns had reported moderate anxiety and burnout and this had affected their ability to care and feel for others and themselves.^2,15^ In a Japanese study, it was noted that interns had a threefold increase in depressive symptoms during the course of their internship.^16^ A Taiwanese study showed that the prevalence of depression in medical interns across three centres was 20.9%.^3^ Other research conducted in Mexico City suggested that 56.0% of junior doctors (including medical interns) had depressive symptoms and 21.0% had reported being diagnosed with major depressive disorder (MDD) during their training.^17^

Possible reasons for this increased risk among the interns included the change in status and responsibility from being a student to that of being a doctor.^8,12^ Interns may also lack interpersonal and communication skills, medical knowledge and experience, making it difficult to adapt to a new role and working environment with higher expectations.^13^ Features associated with poor well-being may include poor relationships with supervisors, other staff and patients, minimal sense of control regarding placements, lack of autonomy in decision-making, poor social support, frequently changing to different departments and a lack of time for personal pursuits.^18,19,20,21^ Several studies have noted that female interns are more at risk of developing depression as opposed to male interns.^3,4,6,11,22,23^

A cross-sectional study of 132 medical doctors (including consultants, registrars, medical officers, community service officers and interns) working at 27 different facilities in the Western Cape conducted by means of a self-administered battery, including the Beck Depression Inventory-2 (BDI-2), was published by Rossouw et al. in 2013. The BDI-2 is a self-reported questionnaire widely used in clinical and research settings for screening and rating the severity of depression.^24^ The study highlighted some of the difficulties that doctors working in South Africa face, which included a heavy work load, long working hours and public system-related difficulties.^25^ The researchers found that interns (*n* = 11) who had the least years of experience had the highest scores on the BDI-2.^25^ Although the features associated with these higher BDI-2 scores were not explored, the topic has been one of ongoing debate and speculation.

Burnout is defined as a persistent, negative, work-related state of mind that is characterised by exhaustion, accompanied by distress, and has negative consequences such as reduced effectiveness, decreased motivation and dysfunctional attitudes and behaviours.^25,26^ There is a complex relationship between burnout and depression. Although burnout in its early stages is confined to a specific work environment, if untreated, it can potentially progress, or be comorbid to depression, which would affect all spheres of a person’s life.^26^

Internship typically involves long working hours, with associated chronic sleep deprivation, which has been associated with numerous negative outcomes including negative mood changes, memory loss, over-optimistic risk taking, increased motor vehicle accidents and needle-stick injuries with possible occupational exposure to HIV.^27^ A study examining occupational stress and burnout among South African medical practitioners concluded that working overtime and poor organisational support, making critical ‘on the spot’ decisions, led to increased symptoms of burnout.^28^ Seeing more than 40 patients per day was also noted to increase symptoms of burnout. A retrospective study conducted among South African doctors noted that 37.3% of doctors had reported feeling burnt out during their internship and that doctors who worked part time were less likely to experience burnout.^29^

MDD has been shown to have many important associated features such as reduced confidence in the performance of clinical tasks, poor-quality patient care and an increase in self-reported medical errors.^18,30^ MDD can potentially be a devastating and incapacitating disorder. If left untreated, it could result in suicide.^31,32^ Rubin has estimated that 300–400 physicians in the US die by suicide each year.^33^ In the US, interns have been shown to have increased thoughts of death in the first 3 months of internship.^34^

There is a lack of research exploring the prevalence of depressive symptoms in interns and the features associated with the development of these symptoms in medical interns in South Africa. Having recognised this gap in the literature, this study aimed to explore these questions in interns employed at a tertiary academic hospital in Cape Town, South Africa.

## Materials and methods

### Study setting and ethical approval

Prior to professional registration as a medical doctor, the Health Professions Council of South Africa requires that all newly qualified doctors undergo a 2-year internship, during which medical graduates can practise their skills under supervision at an accredited institution.^27,35^ Ethical approval for the project was obtained from the Human Research Ethics Committee of the Faculty of Health Sciences at University of Cape Town and ethical clearance was granted for the study by Groote Schuur Hospital (GSH) where the study was conducted. GSH is a large tertiary academic health facility situated in the Western Cape.^36^ The hospital has approximately 90 medical interns who rotate through multiple medical and surgical specialties.

Potential ethical problems included protecting the confidentiality of the participants, ensuring that all participants have access to convenient and appropriate mental health care services and that participants receive appropriate feedback.

### Research design

This study was a cross-sectional observational study. All 91 interns employed at GSH between January 2017 and May 2018 were sent an email invitation to participate in the study. During the meeting, interns were introduced to the study and given a consent form, a demographic questionnaire, a BDI-2 and a related questionnaire (see details below) to complete. All interns first read the aims of the study and that participation in the study was voluntary and confidential and then gave written informed consent if they opted to enrol. There were no exclusion criteria.

### Statistical analysis

All analyses were completed using SPPS version 25. The threshold for statistical significance (*α*) was set at 0.05, unless otherwise noted. For each analysis described below, the appropriate effect size was calculated.

Descriptive statistics were generated for all variables to ensure normality of distributions. Medians and interquartile ranges were used to describe continuous variables and proportions for categorical variables. A series of Mann–Whitney *U* tests compared BDI-2 scores across various demographic and associated features, because assumptions for parametric testing were not upheld. Thereafter, a backwards linear regression analysis was performed to determine whether significant variables from the univariate analyses predicted depressive symptomology.

Beck Depression Inventory-2 scores were coded as categorical variables. Sex was coded as a categorical variable, having two levels (male and female). All other predictor variables were coded having two levels (yes and no). Dummy coding was applied to the variable Burnout, such that ‘not at all burnout’ was the reference group.

### Sampling and materials

The demographic questionnaire was designed to obtain demographic characteristics of the interns as well as information about features commonly associated with depression. This questionnaire was drawn up by the research team for the purposes of this study and is not a validated tool that has been used in other settings.

The demographic questionnaire obtained information about participant’s age, gender, marital status, and the year of their internship. Information regarding features associated with depression included (1) a prior diagnosis of depression or a mental health illness, (2) duration of treatment, (3) family history of depression, (4) previous or current suicidal ideation or attempts and (5) substance use history as well as the frequency and duration of use. Other commonly noted consequences of depression, as noted in the literature, were also asked about. This included the desire to emigrate or leave clinical medicine and the presence of other hobbies or pastimes were also asked about, for example, exercise, spending time with friends or family, meditation or participating in religious or spiritual activities.

Because of the strong association between burnout and depression, a Likert-scale question regarding the subjective feeling of burnout was asked. Participants were asked to rate their subjective feelings of burnout on a scale including ‘not at all burnt out’, ‘slightly burnt out’, ‘moderately burnout’ or ‘severely burnt out’.

Whilst the study was largely quantitative in nature, at the end of the questionnaire the participants were given an opportunity to comment on any other features that they felt would impact on their mental health. This introduced a very small qualitative component with regard to the interns’ experience of internship.

The BDI-2 measures the behavioural manifestations of depression and consists of 21 symptoms or clinical features of depression with various statements rated on a scale of 0–3.^24^ A score of zero indicates the absence of a symptom and a score of 3 indicates its greatest severity. It is important to note that the BDI-2 has not been validated in the South African context but is a widely used psychometric measure of depression severity in clinical and research settings.

A total score of below 14 on the BDI-2 would indicate minimal self-reported symptoms of depression. A total score of between 14 and 19 would indicate mild self-reported features of depression and a score between 20 and 28 would indicate moderate self-reported symptoms of depression, while a score greater than 28 would indicate severe self-reported symptoms of depression. A self-reported score of 14 or above was considered significant for the purposes of this study.

## Ethical consideration

Prior to professional registration as a medical doctor, the Health Professions Council of South Africa requires that all newly qualified doctors undergo a 2-year internship, during which medical graduates can practise their skills under supervision at an accredited institution.^27,35^ Ethical approval for the project was obtained from the Human Research Ethics Committee of the Faculty of Health Sciences at the University of Cape Town and ethical clearance was granted by GSH where the study was conducted. GSH is a large tertiary academic health facility situated in the Western Cape.^36^ The hospital has approximately 90 medical interns who rotate through multiple medical and surgical specialties. Potential ethical problems included protecting the confidentiality of the participants, ensuring that all participants have access to convenient and appropriate mental health care services and that participants receive appropriate feedback. A meeting was arranged for interns to participate in the study which took place on the 12th of April 2018. All participants were given a consent form to sign. This consent form highlighted that participation was voluntary, that participants could withdraw from the study at any time and that there would be no financial remuneration in return for their participation in the study. To ensure confidentiality and anonymity, no identifying data were asked on the ‘Demographic and Related Questionnaire’ nor on the BDI-2 and equally consent forms were collected separately to other questionnaires. Each participant received an information pack detailing the symptoms of burnout and depression and pathways for accessing mental health care services should they feel they require it. The contact details of the researchers were provided should participants have any questions or want a referral to facilitate pathways to further care. Feedback will be given to all participants following publication.

## Results

### Depression during internship

Out of a possible 91 interns invited to join in the study, 57 (62.6%) agreed to participate. Three participants returned incomplete questionnaires and were consequently excluded from the study. This led to a final participation rate of 59.3%. Interns were on average 25.4 (s.d. = 1.4) years old, 65.0% (*n* = 35) were female and 35.0% (*n* = 19) were male; 48.0% (*n* = 26) were single, whereas 33.0% (*n* = 18) were married, and 50.0% (*n* = 27) were in their first year of internship (the remaining 48% [*n* = 26] were in their second year). One intern did not specify their internship year.

Thirty-two interns (59.2%) had a BDI-2 score of below 14, indicating minimal self-reported symptoms of depression, 9 (16.7%) had scores between 14 and 19 which indicated mild self-reported symptoms, 6 (11.1%) had scores between 20 and 28 which indicated moderate self-reported symptoms and 7 (12.9%) had scores greater than or equal to 29 which indicated severe self-reported symptoms of depression. The mean BDI-2 score was 14 (s.d. = 10.8) and the median was 11, with the range being 0–47.

Based on the demographics, 19 female interns (54.2%) reported symptoms of depression (indicated by BDI-2 scores of 14 or above) with 11 female interns (31.4%) within this subgroup who had moderate to severe self-reported depressive symptoms (as indicated by BDI-2 scores of 20 or above). Three male interns (15.8%) reported symptoms of depression with BDI-2 scores of 14 and above. Within this group, 2 male interns (10.5%) reported severe symptoms of depression as indicated by a total BDI-2 score of 29 or greater.

Regarding BDI-2 scores, 22.2% (*n* = 6) of first-year interns experienced moderate to severe self-reported symptoms of depression as opposed to 26.9% (*n* = 7) of second-year interns who reported the same (see Table 1).

**TABLE 1:** Demographic characteristics and associated Beck Depression Inventory 2 scores.

Variable	Minimal self-reported depressive symptoms (*n*)	BDI-2 score < 14 (%)	Mild self-reported depressive symptoms (*n*)	BDI-2 score ≥ 14–19 (%)	Moderate self- reported depressive symptoms (*n*)	BDI- 2 score ≥ 20–28 (%)	Severe self-reported depressive symptoms (*n*)	BDI- 2 score of > 29 (%)
Total sample population (*n* = 54)	32	59	9	16.6	6	11.1	7	12.9
Female (*n* = 35) 65%	16	45.7	8	22.8	6	17.1	5	14.2
Male (*n* = 19) 35%	16	84.2	1	5.3	0	0	2	10.5
First-year interns (*n* = 27) 50%	17	63	4	14.8	3	11.1	3	11.1
Second-year interns (*n* = 26) 48%	13	50	6	23.1	3	11.5	4	15.4

BDI, Beck Depression Inventory.

Antidepressants had been prescribed to 8 interns (14.8%) during their internship, but only 4 (7.4%) reported taking them at the time of assessment. These antidepressants had been prescribed by general practitioners for three interns and by a psychiatrist by the remaining five. The eight interns who were prescribed antidepressants during their internship had significantly higher BDI-2 scores compared with the rest of the interns (median = 26.5 vs. 11; *U* = 65, *p* = 0.003, *r* = 0.39). The four interns currently using antidepressants did not have significantly higher BDI-2 scores compared to the rest of the interns (median = 20 vs. 11; *U* = 60, *p* = 0.200, *r* = 0.18).

Also of note at the time of data collection, 3 interns (5.5%) were seeing a psychologist and using antidepressants simultaneously. Three interns (5.5%) reported seeing a psychologist without being on antidepressants and one intern (1.8%) reported taking antidepressants without seeing a psychologist.

Worryingly, six interns reported that they had thought about taking their own life and this correlated with higher BDI-2 scores. Almost every one of the interns reported subject feeling of burnout as well as having too little time to attend to their own or significant others’ needs. Eight interns reported using substances to cope with the stressors of internship (see Table 2).

**TABLE 2:** Features associated with depressive symptomatology.

Variable	*N*	Median	IQR	*U*	*p*	ESE
**Gender**				178	0.005	0.38
Male	19	7	3–11	-	-	-
Female	35	15	9–25	-	-	
**Considered taking own life**				53	0.010	0.34
Yes	6	28.5	15.5–41	-	-	-
No	48	11	5–16.75	-	-	-
**Substance used to cope**				98.5	0.036	0.28
Yes	8	24	8–38	-	-	-
No	46	11	5–16.5	-	-	-
**Lack of time for relationships**				24.5	0.008	0.34
Yes	50	11.5	5.75–21.75	-	-	-
No	4	3.5	0.75–6.25	-	-	-
**Emigration**				155.5	0.003	0.40
Yes	37	15	8.5–25	-	-	-
No	17	5	3–11.5	-	-	-
**Leaving medicine**				113.5	< 0.001	0.49
Yes	37	16	9.5–26.5	-	-	-
No	16	5.5	3.25–10.75	-	-	-

Note: Self-reported depressive symptom severity represented by BDI-2 total scores. ESE, effect size estimate, in this case, *r.*

IQR, interquartile range.

### Depression or psychiatric illness and pathways to care prior to internship

Eight interns (14.8%) had previously been diagnosed with depression prior to their internship, and an additional 5 interns (9.3%) with a psychiatric illness other than depression (e.g. bipolar disorder, generalised anxiety disorder, eating disorder, attention deficit disorder). Five of the 8 interns saw a therapist for their depression, and all 8 had previously been prescribed antidepressants (median = 26.5 vs. 11; *U* = 85.5, *p* = 0.014, *r* = 0.33). The 6 interns who saw a therapist for their depression had significantly higher BDI-2 scores compared to the rest of the interns (median = 22 vs. 11; *U* = 62, *p* = 0.022, *r* = 0.31). Of the 8 interns previously diagnosed with depression, only 3 interns (5.8%) were currently on antidepressant treatment.

### Variables associated with Beck Depression Inventory 2 scores

Female interns had significantly higher BDI-2 scores compared to male interns (median = 15 vs. 7; *p* = 0.005). Interns who reported suicidal thoughts during their internship, those who used substances to cope during their internship, those who were considering emigration because of current working conditions and those who were considering leaving medicine because of their internship had significantly higher BDI-2 scores (all *p*-values < 0.036; see Table 2).

Seven (12.9%) interns cited workplace bullying and not being treated with respect as a major problem and contributing factor to their mental state. It is noted that three interns who cited work bullying as a source of stress also reported moderate to severe symptoms of depression. Five interns (9.2%) reported that long working hours and the associated sleep deprivation were associated with their feeling depressed; however, only two interns who cited this had mild self-reported symptoms of depression.

There was a statistically significant difference in BDI-2 scores among participants with different subjective levels of burnout (*F* [3, 53] = 5.77, *p* = 0.002). Post-hoc comparisons revealed that interns reported being ‘moderately burnt out’ (median BDI = 15) had significantly higher BDI-2 scores compared to interns who reported being not at all burnt out’ (median BDI = 1) or ‘slightly burnt out’ (median BDI = 9; *p* = 0.019 and *p* = 0.044, respectively). Further, interns who reported themselves as ‘severely burnt out’ (median BDI = 25) had significantly higher BDI-2 scores compared to interns who were not burnt out or slightly burnt out (*p* = 0.002 and *p* = 0.001, respectively).

### Regression analysis

A backwards linear regression model was conducted to assess the combined association between significant variables found in the univariable analyses and depressive symptomology (see Table 3).^1^ The variables ‘Gender’, ‘Emigration’ and ‘Wanting to Leave Medicine’ were entered in the first block, and ‘Burnout’ in the second block. None of the data violated assumptions of independence. Some of the independent variables in the regression were highly correlated (see Table 3).

**TABLE 3:** Correlations between outcome and predictor variables.

	BDI-2	Gender	Emigration	Leaving medicine	Burnout (slight)	Burnout (moderate)	Burnout (severe)
BDI-2 (outcome)	1.00	−0.296*	0.326*	0.455**	−0.341*	0.182	0.365*
Gender	-	1.00	−0.190	0.038	0.152	−0.010	−0.269*
Emigration	-	-	1.00	0.341*	−0.270*	0.151	0.352*
Leaving medicine	-	-	-	1.00	−0.560**	0.211	0.337*
Burnout (slight)	-	-	-	-	1.00	−0.581**	−0.415*
Burnout (moderate)	-	-	-	-	-	1.00	−0.367*
Burnout (severe)	-	-	-	-	-	-	1.00

Note: Values presented are Pearson’s correlation coefficients.

* *p* < 0.05;

** *p* < 0.001.

BDI-2, Beck Depression Inventory 2.

In Model 1, ‘Emigration’ was the least significant predictor of BDI-2 scores (*p* = 0.689) and was removed from Model 2. Model 2 was statistically significant (*p* < 0.001) and indicated that ‘Wanting to Leave Medicine’ and ‘Burnout’ were the only significant predictors of BDI-2 scores. Overall, the initial regression model explains 36% of the variance in participants’ BDI-2 scores. As the variable ‘Gender’ was not a significant predictor in Model 2, it was removed and a final backwards regression was run.

Overall, the final model that included Wanting to Leave Medicine and Burnout explained 33% of the variance in participants’ BDI-2 scores (*R* = 0.617, Adjusted *R*² = .329, *F* [4,52] = 7.37, *p* < 0.001). The final regression equation was: *y* = −7.37 + 9.04 (Leaving Medicine) + 13.74 (Burnout slight) + 16.78 (Burnout moderate) + 20.15 (Burnout severe). The beta coefficients indicate that severe burnout was the strongest predictor of BDI-2 scores (see Table 4).

**TABLE 4:** Table of regression analysis to examine features associated with depression and their Beck Depression Inventory 2 scores.

Variable	*B*	s.e.	Beta	*T*	*p*
**Model 2**					
Constant	−5.12	5.96		−0.86	0.395
Gender	−4.94	2.65	−0.22	−1.87	0.069
Leaving medicine	10.08	3.18	0.44	3.17	0.003*
Burnout (slight)	13.21	5.62	0.61	2.35	0.023*
Burnout (moderate)	15.31	5.41	0.69	2.83	0.007*
Burnout (severe)	17.31	5.77	0.66	3.00	0.004*
**Final regression analysis**					
Constant	−7.37	5.99		−1.23	0.224
Leaving medicine	9.04	3.21	0.392	2.82	0.007*
Burnout (slight)	13.74	5.76	0.635	2.39	0.021*
Burnout (moderate)	16.78	5.49	0.751	3.06	0.004*
Burnout (severe)	20.15	5.70	0.772	3.53	0.001*

s.e., standard error.

* *p* < 0.005.

**FIGURE 1:**
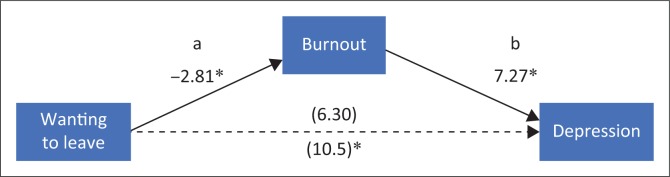
Mediation analysis.

A mediation analysis was conducted (see Figure 1) to determine whether Wanting to Leave Medicine predicts BDI-2 scores indirectly through Burnout. To conduct this test, Burnout was dichotomised (Level 1: not at all and slight; Level 2: moderate and severe). The Sobel test was significant (Sobel *Z* = −1.97, *p* = 0.049), indicating that Wanting to Leave Medicine predicts BDI-2 scores through Burnout. Therefore, Burnout is the sole predictor of depressive symptomology.

## Discussion

The primary aim of this study was to determine the associations between demographic data, previous history of depression, previous or current treatment for depression and substance use and the self-reported severity of depressive symptomology of medical interns at GSH. The secondary aim is to identify risk factors for the development of depressive symptoms during internship.

In our sample, 22 (40.7%) out of the 54 participating interns showed features of depression (indicated by a BDI-2 score greater than 13). This is comparable to international literature, showing that the symptoms of depression among interns far exceeds that of the general population of South Africa which at the time of South African Stress and Health Study was 9.7%.^14^

Female interns have been shown to have higher BDI-2 scores when compared to male interns.^3,4,6,11,22,23^ Our findings are similar in that 54.0% of female interns reported a higher prevalence of depressive symptoms compared to only 15.0% of male interns.

Contrary to the literature, we found that first-year interns reported less symptoms of depression compared to those in their second year. In contrast, Rossouw et al. showed that the BDI-2 scores were negatively correlated to years of experience.^25^ In this study, when severity of depression is examined, it is noted that three first-year interns had BDI-2 scores of 29 or greater, indicating severe depression, as opposed to 4 second-year interns who had scores of 29 or greater. Seeing that our sample size is relatively small and from one centre in the Western Cape, we would require further research to see if second-year interns have higher BDI-2 scores than first-year interns.

In our study, risk factors associated with the development of symptoms of depression included female gender and having a prior diagnosis of a mental illness and having the subjective experience of being moderately or severely burnt out. Being in psychotherapy at the time of the study was associated with higher BDI-2 scores. One could speculate that the five interns who had experienced more severe symptoms of depression then sought help from a psychotherapist. Whilst the research shows this association, it cannot be said whether it could be causative or consequential.

Interestingly, the four interns who were currently taking antidepressants did not have significantly higher BDI-2 scores when compared to the rest of the interns. This may indicate the importance of early diagnosis, continued support and adherence to medication. Interns who were previously on antidepressant treatment during internship could have potentially had an improvement in their depressive symptoms which could have led them to stopping treatment. It is concerning that whilst 24.1% (*n* = 13) of our total sample reported moderate to severe symptoms of depression, 7.7% (*n* = 1) of this subgroup were receiving psychotherapy only and 2 interns (15.4%) were receiving a combination of psychotherapy and antidepressants. This potentially means 76.9% (*n* = 10) of the interns who had moderate to severe symptoms of depression were not on any treatment presently. This represents a large treatment gap. This has been shown in other studies where interns have a preference to manage problems on their own, believing that stress is a part of the job, fearing the stigma associated with seeking help, lack of convenient mental health care services and possibly the fear of being viewed as impaired.^33,37,38,39,40^

More than 90.0% (*n* = 50) of all participants had commented that because of lack of time, both their relationships and personal pursuits were being compromised. These results are concerning as reduced engagement with hobbies and loved ones also predispose to burnout and depression.^41^ Fifty-one participants (96.0%) reported the subjective feeling of burnout during their internship. The prior study by Rossouw et al. indicated that 76.0% of their sample displayed some degree of burnout. This is considerably high in our study population. The important difference between this and the Rossouw et al. study was that their study also included senior medical officers and specialists.

Thirty-nine (68.5%) interns reported wanting to emigrate because of current or possible future working conditions. This is a great concern considering the scarcity of doctors within South Africa.^27,42^ It was noted by Mayosi et al. that approximately 30.0% of South African medical doctors had emigrated to other countries and approximately 58.0% were considering emigrating. The desire to emigrate because of current or future working conditions is much higher within our study population. This can lead to a potentially large loss of resources for South Africa, especially considering the high cost of training doctors who would then not be practising in the country.^42^ Of note, interns with higher BDI-2 scores were also more likely to want to leave clinical medicine.

During their internship, 6 interns (11.1%) reported ‘thinking about taking their own life’. This symptom of depression was noted to correlate with higher BDI-2 scores than those who did not have suicidality. It was noted by Goldman et al. and Guille et al. that there is an increase in suicidal ideation in the first 3 months of internship.^34,43^ Interestingly, in our study, it was noted that the majority (*n* = 5) who had suicidal ideation were in their second year of internship and only one was in their first year. This highlights the importance of identifying interns who could be at greater risk to experience serious suicidality and to continually provide support for interns throughout their internship.

Eight interns (14.8%) reported using substances (prescription medication and illicit substances) to help them cope with the stresses of internship. Three interns did not specify which substances they used and 4 reported using anxiolytics or hypnotics to help them to sleep and one admitted to using cannabis and diazepam. Eleven interns (20.3%) used alcohol as a coping mechanism and the use of alcohol and substances was associated with significantly higher BDI-2 scores. The median BDI-2 score for interns who engaged in substance use was 24. The use of substances in this group was greater than anticipated. In a previous study conducted in Mexico City, 21.0% of interns were at risk for problematic alcohol use and 2.0% of interns consumed recreational drugs once per month.^17^ It is also possible that these interns may be using alcohol and other substances to manage their depressive symptoms.

Workplace bullying has been shown to be a risk factor for the development of burnout and depression in international studies.^18,19,20,21^ This was replicated in this study where seven interns reported workplace bullying as negatively impacting their mental state. Other features included, staff shortages and long work hours as contributing to decreased well-being, as noted by five interns.

The subjective feeling of burnout may be a marker for identifying interns with depression or at risk of developing depression. There may be less stigma associated with the term than depression. This is important to note that further research in the field of burnout and its prevention can help prevent the development of depressive symptoms in interns.

## Limitations

There are several limitations to this study. As this is a cross sectional study, causality cannot be determined. Ideally, a prospective study measuring the BDI-2 scores at the beginning of internship and following up the scores during the course of internship could then determine whether there is an increase in BDI-2 scores as time progresses. Because the demographic and related questionnaire and the BDI-2 were self-administered, there could be an over- or under-reporting of symptoms. Whilst the BDI-2 is considered by many to be the ‘gold standard’ instrument in the measurement of depressive symptoms, its use has not yet been validated in a South African context.

Because of the small sample size, it is not possible to determine bidirectionality between burnout and depression and wanting to leave medicine. More robust studies, with larger sample sizes, would be required for this. Although this study focused on depression and not burnout, because of the suggested strong association between the two, it is highly recommended that future research uses more established questionnaires to describe and measure burnout. Our study used a single Likert-style question to measure subjective burnout, so we are cautious in making the association between depression and burnout.

The study was conducted in a single tertiary hospital in the Western Cape and the results may not be generalisable to the rest of the country or even to the province.

## Conclusion

This study represents a small subset of interns and the challenges that they face in South Africa. There is an indication that interns employed at GSH are at risk for developing depression. The most significant factor associated with reported symptoms of depression was a subjective feeling of being moderately to severely ‘burnt out’. Some features to look at would and educating senior staff about the effect that workplace bullying has on their junior colleagues. It is noted that some interns do have maladaptive coping strategies including alcohol and substance use. Also concerning is the number of interns with suicidal ideation. Onsite counselling may also help provide more support to interns. Screening of interns in need of mental health services may help to also provide better support to interns at risk of developing depression and burnout. Larger studies involving more interns across all provinces are needed to adequately assess the challenges that interns face and how to best assist them so that they may develop into well-adjusted clinicians who better serve the communities they treat.

## Footnotes

1 Although the variables *intern taking own life, using substances to cope* and *lack of time for relationships* were significantly associated with BDI-2 scores in the univariate analyses, they were not entered into the linear regression because of the limited number of participants who answered yes to these questions.
